# A Financial Market Model Incorporating Herd Behaviour

**DOI:** 10.1371/journal.pone.0151790

**Published:** 2016-03-23

**Authors:** Christopher M. Wray, Steven R. Bishop

**Affiliations:** Department of Mathematics, University College London, London, United Kingdom; Universiteit Gent, BELGIUM

## Abstract

Herd behaviour in financial markets is a recurring phenomenon that exacerbates asset price volatility, and is considered a possible contributor to market fragility. While numerous studies investigate herd behaviour in financial markets, it is often considered without reference to the pricing of financial instruments or other market dynamics. Here, a trader interaction model based upon informational cascades in the presence of information thresholds is used to construct a new model of asset price returns that allows for both quiescent and herd-like regimes. Agent interaction is modelled using a stochastic pulse-coupled network, parametrised by information thresholds and a network coupling probability. Agents may possess either one or two information thresholds that, in each case, determine the number of distinct states an agent may occupy before trading takes place. In the case where agents possess two thresholds (labelled as the finite state-space model, corresponding to agents’ accumulating information over a bounded state-space), and where coupling strength is maximal, an asymptotic expression for the cascade-size probability is derived and shown to follow a power law when a critical value of network coupling probability is attained. For a range of model parameters, a mixture of negative binomial distributions is used to approximate the cascade-size distribution. This approximation is subsequently used to express the volatility of model price returns in terms of the model parameter which controls the network coupling probability. In the case where agents possess a single pulse-coupling threshold (labelled as the semi-infinite state-space model corresponding to agents’ accumulating information over an unbounded state-space), numerical evidence is presented that demonstrates volatility clustering and long-memory patterns in the volatility of asset returns. Finally, output from the model is compared to both the distribution of historical stock returns and the market price of an equity index option.

## Introduction

For more than a decade, herd behaviour [1, 2] in financial markets has been the subject of much research [3–7], in parallel with research investigating the phenomenon of stock market crashes [8–10], and the identification of certain stylised features of financial market data (see the excellent reviews by R. Cont [11] and P. Bouchaud [12]). Recent extraordinary market events [13, 14], reviewed by Cincotti, et. al [15], demonstrate that herd behaviour can have material consequences for investors, and regulators, alike [16]. While identifying and estimating the impact of herd behaviour on financial markets remains a challenge, technological and market developments have increased the potential for herding to arise. For instance, investor sentiment mined from social media [17–19], and the availability of data sets quantifying collective behaviour [20, 21], have the potential to facilitate both intentional and spurious herding (using the terminology of Bikhchandani and Sharma [3]). Evidence suggests [22] that such data are already being used by global asset managers. Furthermore, in a report commissioned by the UK government [23], herd behaviour is identified as a possible consequence of high-frequency trading, which constitutes a significant and growing proportion of trading activity [24].

Previous attempts at understanding the dynamics of financial markets have primarily focused on accurately describing the observed data using time-series analysis, or purely statistical methods. It is well-documented that price returns of financial assets exhibit significant deviations from the Gaussian model [11, 25], which has resulted in a plethora of alternative representations. Models such as *α*-stable distributions [26], generalised hyperbolic models [27], generalised autoregressive conditional heteroskedasticity (GARCH) models [28] and stochastic volatility models [29] attempt to account for features, such as high kurtosis, long-memory [30] and volatility clustering [31, 32] which are inconsistent with Gaussian behaviour. In addition, various studies [33–35] have demonstrated financial returns may be better described by a power law with exponent, *α*, outside of the stable-Lévy regime of 0 < *α* < 2.

Alternative descriptions of financial market dynamics using agent-based models [4, 7, 36, 37] have contributed to the qualitative understanding of asset price processes (for an excellent review of this area, see the report by Chakraborti [38]), but have not yet achieved widespread acceptance amongst market participants. Feng, et al [39] suggests one reason for the lack of acceptance of agent-based financial market models is the insufficient quantitative accuracy of such models—and the paper advocates combining an agent-based approach with classical stochastic modelling in order to link microscopic agent behaviour with macroscopic phenomena [40]. Following this line of enquiry, we develop a new archetypal probabilistic interacting agent (trader) model to describe price fluctuations in a market for a single asset where agents stochastically accumulate information, up to some threshold (the information threshold) prior to initiating a trade. The information accrual of agents is modelled using a discrete state-space enumerated by integers. Under this assumption, agent information thresholds correspond to maximal and minimal integers that define the information state-space. In this study, we consider both bounded and unbounded information state-spaces, as described in the methods section. In line with Feng et.al [39], we model only those agents in a market considered to be technical traders [41] as a result, all agents may engage in either buying or selling of the asset, determined by a binary random variable (modelling the result of some decision process) taking the values +1 for a buy and −1 for a sell with equal probability, prior to agents reaching their information threshold. Under this assumption, the average demand is 0, and therefore the market fluctuates around equilibrium.

Agents are considered to have recourse to two sources of information: private information (of a level unknown to other agents) and public information inferred probabilistically from the actions (trades) of other agents. It is assumed that agents combine information from both sources additively when determining if their information threshold is reached. Therefore, when one agent reaches their information threshold and instigates a trade—this information is (probabilistically) incorporated into the information set of other agents who observe the market impact of the trade on the asset price. We define the event of an agent reaching their information threshold immediately after observing the trade of some other agent, as a cascade—and for notational convenience—define the event of a single agent reaching their information threshold, but inducing no other agents to their respective information thresholds, as a cascade of size one. When agents are brought to their information threshold as part of a cascade, their individual demand is taken to be equal to that of the agent that initiated the cascade—and the aggregate demand is considered to be excess demand that changes the market price according to a given price impact function. In contrast to economic models, we do not model the decision process, or its optimality, explicitly.

In order to model this situation, we utilise a network of stochastic pulse-coupled integrate and fire oscillators [42], in which oscillators are identified as agents. The integrate phase, bounded by the information thresholds, is identified with agent information accrual, and the firing phase is identified with agent trading. Instantaneous pulse-coupling is used to capture implicit agent interaction, which differentiates our model from those that employ continuous Ising-like coupling [43], the latter being difficult to justify in a financial markets context where, for fiduciary, competitive and regulatory reasons, agents are not expected to intentionally and directly interact for prolonged durations. In line with model parsimony, different agents may be endowed with differing information thresholds although they remain constant for each agent.

Under the condition that agent decesions are economically rational, our model can be interpreted in the economic context of rational herding via (probabilistic) informational cascades [44, 45]. An informational cascade is said to occur when agents obtain information by observing the actions of others and who may then (optimally) decide to act against their own private information as a result. For instance, an agent whom would otherwise arrive at a decision to sell an asset after accruing their private information may be led to instead buy the asset, upon observing enough investors buying the security, through the impact on asset price. Although the notion of an informational cascade is frequently encountered in economic contexts, it is of general applicability to scenarios consisting of agents that are subject to social learning, while possessing a limited action space (Ellis and Fender [46] provide an example of the use of informational cascades in the context of political regime change).

In terms of agent behaviour, our study differs from similar existing analyses [39], by modelling both sequential asynchronous agent trading (cascades of size one), and simultaneous synchronous trading (cascades of size greater than one) which may facilitate application to scenarios involving markets consisting of a subset of high-frequency traders. Furthermore, the method used here to model agent informational accrual allows for the case when agent information accumulates over an unbounded region, which may realistically account for the times when agents withdraw from the market, or may be otherwise unwilling to trade.

It is important to note that here we are primarily concerned with the quantity of information accrued, and not with the quality of the information. In particular, we do not stipulate how agents obtain their private information, nor their decision making processes. Although not incorporated into the current study, our model does not preclude the scenarios of correlated private agent information, unequal weighting of private and public information, or that of agent decisions being governed by a well-defined mechanism (such as adherence to quantitative trading rules).

Phenomenologically, our model is related to the class of stochastic volatility models known as multifractal and multiplicative cascade models [47–50], although in contrast, ours is based upon agent (trader) interactions and information thresholds, and not on fixed heterogeneous time scales. The network used here comprises of *N* vertices, which represent the *N* agents, and the model may be parametrised by information threshold, *K*, and network pulse-coupling probability, *Kq*/*N* (with *q* > 0 a network coupling probability parameter) when all agents have identical information thresholds. In this case, as *N* tends to infinity, this model is known to transition from a quiescent state to a synchronised herd-like state as *q* surpasses one. By computing the logarithmic asset price-returns (log-returns) arising from our model, we demonstrate that a number of stylised facts concerning financial returns can be reproduced. Certain features of empirical data, such as long-tailed and power law distributed price returns, are reproduced. The so-called (Black-Scholes [51] implied) volatility smile [52], obtained from the price of index option contracts [53], is approximately also recovered. The presentation of our results begin by establishing a number of mathematical properties of the underlying models used to describe our financial market model. In the case where agents’ information accrual occurs over a bounded state-space (labelled as the finite state-space model) and with *K* = 1, an asymptotic expression for the cascade probability is derived, while for *K* ≥ 1, comparison with the negative binomial distribution enables the functional form of price return standard deviation (also known as volatility in the lexicon of financial markets) to be expressed in terms of *q*. Substantial numerical simulations are used to demonstrate volatility clustering, and a long-memory pattern in asset return volatility autocorrelation, occurs in the case where agents’ information accrual occurs over an unbounded state-space (labelled as the semi-infinite state-space model). Our results suggest that a range of stylised facts can be attributed to how agents process and accumulate information, and that explicit expressions of asset return volatility and kurtosis can be obtained in terms of information thresholds and network coupling probability.

## Results

### Stochastic cascade process and cascade size

**Fig 1:**
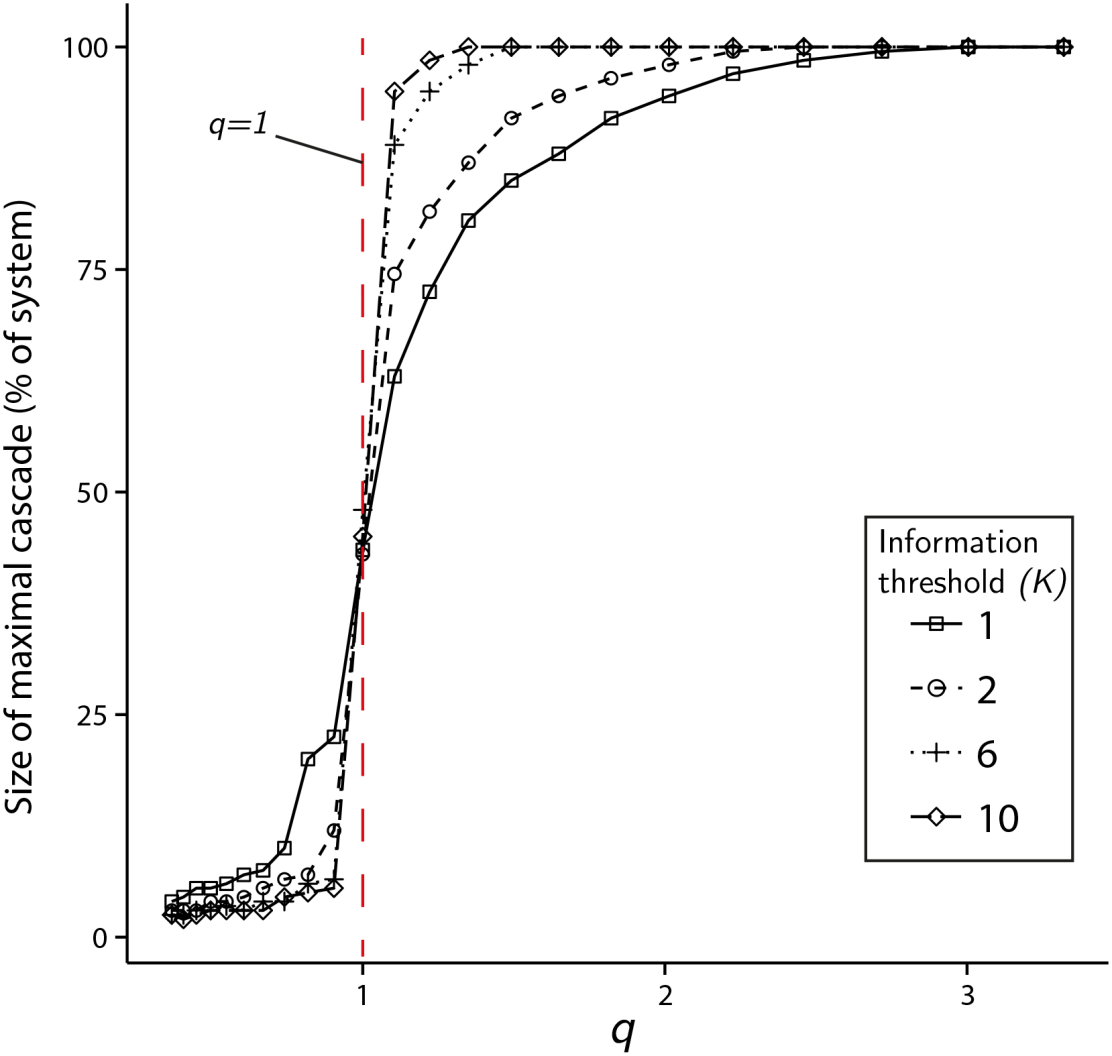
Maximal cascade size of semi-infinite state-space models. Maximal cascade size, expressed as a fraction of system size (*N* = 1000) in the case of agents possessing identical information thresholds, *K*. For each *K* = 1, 2, 6, 10 and each *q* ∈ [0.5, 3.5], the system is simulated until 10^7^ cascades are produced. The magnitude of the largest cascade size, disregarding sign, is then plotted. As *K* increases, the transition to the large cascade regime (*q* > 1) becomes increasingly abrupt.

In the finite state-space model, with *N* agents and a constant information threshold of *K* for all agents (described in the methods section), the information accrual of agents is represented by a random walk on the finite set of integers {0, 1, 2, …, 2*K*}. Recall that state *K* represents a neutral information state, while states 0 and 2*K* represent information threshold states. When an agent’s information accumulation level reaches either one of the threshold states, it is permitted to instigate a trade, which may probabilistically induce other agents to carry out the same trade as part of a cascade. For the semi-infinite state-space model, agents accumulate information represented as transitions on the unbounded set {…, *K* − 2, *K* − 1, *K*}, with the information threshold state represented as *K*. Similar to the finite-state model, when an agent reaches their information threshold, the remaining agents can be induced into a cascade, the size of which depends upon *K* and *q*. In both the finite state-space and semi-infinite state-space cases, the total number of agents that are induced to their information threshold as part of a cascade is referred to as the cascade size, and is denoted by the variable *m*. When such a system is simulated, the size of the *i*-th cascade is denoted as *m*_*i*_, and the sequence {*m*_*i*_}_*i* ≥ 1_ is referred to as the stochastic cascade process, or similarly as the (*K*, *q*) process. Fig 1 shows the maximal cascade size magnitude obtained during simulations of semi-infinite state-space models, each consisting of agents with identical information thresholds at *K*. Results for *K* = 1, 2, 6, 10 are shown varying with *q*. The corresponding results for the finite state-space model is qualitatively similar.

### Cascade distribution of the *K* = 1 finite state-space model

Here, agents are homogeneous by virtue of possessing identical information thresholds, represented by *K*, and accumulate information over an identical bounded state-space. In this case, the agent information threshold is considered part of the system specification, for notational convenience. The model details are recounted in the methods section.

The *K* = 1 system represents a special case as it can be most readily analysed using standard statistical methods. In this case, the system has three states: two firing states and a rest state. This implies that after each cascade event all agents will occupy the rest state, unconditional on their state prior to the cascade event and the cascade size. The system then repeats in this way. As a result, the cascade sizes can be considered to be independent and identically distributed statistical random variables. For *K* > 1, the system can be said to possess memory, because cascade sizes depend upon the outcome of previous cascades due, in part, to the distribution of agents among the system states generally differing after each cascade event.

When a cascade is initialised, the number of agents that are subsequently induced to fire is governed by a stochastic process. Furthermore, during the course of a single cascade, agents can only be induced to the firing state at which the cascade is initialised, as agents either transition closer to the firing state, or do not transition at all. We proceed by breaking the development of an arbitrary cascade into discrete levels. Let *X*_0_ = 1 represent the initial firing, and *X*_*k*_ represent the number of agents that fire at the *k*-th level. The total number of agents that have fired by level *n* is written as

$$\begin{array}{c} F_{n}=\sum_{k=0}^{n}X_{k}. \end{array}$$ (1)

Once agents are induced to the firing state, for a given level of the cascade, they fire serially and then enter a refractory state—reducing the number of agents available to be induced to the firing state at the next level. Hence,

$$\begin{array}{c} X_{0}=1,X_{k}=\sum_{i=1}^{X_{k-1}}Y_{i,k} \end{array}$$ (2)

where *Y*_*i*, *k*_ is a binomial random variable given by

$$\begin{array}{c} Y_{i,k}∼\text{Bin}\left(N-F_{k-1}-\sum_{j=0}^{i-1}Y_{j,k},p\right) \end{array}$$ (3)

and *Y*_0,*k*_ = 0. The cascade stops at some level *T* < *N*, with

$$\begin{array}{c} T=\min\{n\;|\;F_{n}=N\;\text{or}\;X_{n}=0\} \end{array}$$

and the cascade size is taken to be *F*_*T*_. The process defined by Eqs (1)–(3) is similar to a Galton-Watson process [54], with the exception that our model is finite (meaning the process always stops) and “offspring” distributions do not satisfy the independence requirement (*X*_*k*_ for *k* > 1 is the sum of dependent binomial random variables).

#### Shrinking *N*-ary trees

**Fig 2:**
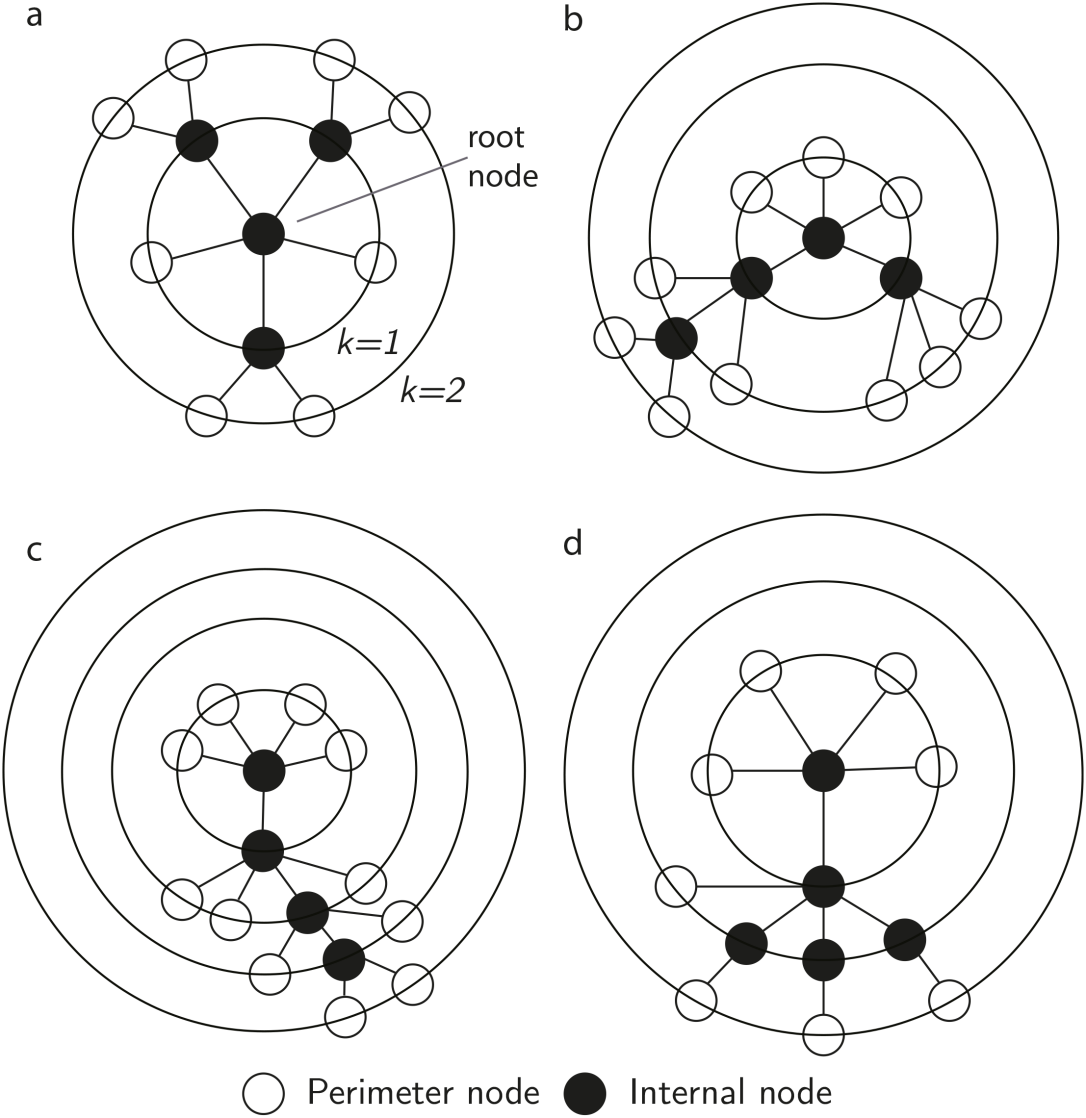
Tree representations of a single arbitrary cascade incorporating dependency between levels. Filled nodes are internal nodes, representing agents induced to the firing state during the course of the cascade. Open nodes are perimeter nodes, representing the unsuccessful attempt of a connected parent node at the preceding level to induce an agent to the firing state. For all panels *N* = 6. a) depicts a cascade of size *m* = 4, with perimeter *Q* = 8, b) *m* = 4 with *Q* = 10, c) *m* = 4 with *Q* = 11, d) *m* = 5 with *Q* = 8.

To obtain an asymptotic expression for the probability of a given cascade size, combinatorial methods are applied to a variant of rooted incomplete *N*-ary trees [55]. A graphical interpretation of the tree-representation of an arbitrary cascade, described below, is presented in Fig 2. Starting with a given single root node (level 0, *X*_0_ = 1), the evolution of a single cascade can be represented exactly by a tree consisting of two types of nodes: internal nodes and perimeter nodes [56]. An internal node, at a given level of the tree, represents an agent induced to the firing state by an agent at the preceding level. A perimeter node represents an unsuccessful attempt, by an agent in a firing state at the previous level, to induce an agent to the firing state. Thus, perimeter nodes are connected to parent internal nodes, and do not produce any further branches. The collection of all perimeter nodes is called the perimeter of the tree, and the size of the perimeter, *Q*, is equal to the number of perimeter nodes. A cascade terminates when the firing state becomes unoccupied—which is represented in the tree as all nodes of a given level consisting of perimeter nodes (which means the tree stops growing). Therefore, a tree consisting of *m* internal nodes, and *Q* perimeter nodes, represents a cascade of size *m*. It follows the probability of a cascade of size *m* can be written in the form

$$\begin{array}{c} P\left(m\right)=\sum_{Q}G\left(m,Q\right)p^{m-1}{\left(1-p\right)}^{Q} \end{array}$$ (4)

where the summation is taken over different values of *Q* that correspond to a single value of *m*, and *G*(*m*, *Q*) is the number of trees consisting of *m* internal, and *Q* perimeter, nodes. When the number of agents remain constant at each level, for instance equal to (*N* − 1), an arbitrary cascade can be modelled using a standard (rooted, incomplete) (*N* − 1)-ary tree. In this case the number *G*(*m*, *Q*) is given by the Fuss-Catalan numbers (generalised Catalan numbers) [57, 58]

G(m,Q)=Q-1(N-1)mm (5)

where *Q* is a 1–1 function of *m* given by

$$\begin{array}{c} Q=m(N-2)+1. \end{array}$$ (6)

When dependence between levels of the tree is taken in to account, according to Eqs (1)–(3), the arity of the tree representing a cascade shrinks monotonically as the cascade progresses (see Fig 2). For example, level 1 consists of a single-level (*N* − 1)-ary tree, while level 2 is a single-level tree, distributed over *X*_1_ root nodes, able to produce up to (*N* − 1 − *X*_1_) internal nodes in total—and so on. For this tree structure we obtain the perimeter size, given the number of internal nodes *m*, as

$$\begin{array}{c} Q=m\left(N-m\right)+\frac{1}{2}{\left(m-1\right)}^{2}-\frac{1}{2}\sum_{k\geq 1}X_{k}^{2} \end{array}$$ (7)

and asymptotically for large *N* the probability of cascade size reduces to,

$$\begin{array}{c} P\left(m\right)∼{\left(2\pi \right)}^{-\frac{1}{2}}m^{-\frac{3}{2}}e^{(1-q)m}q^{m-1}. \end{array}$$ (8)

**Fig 3:**
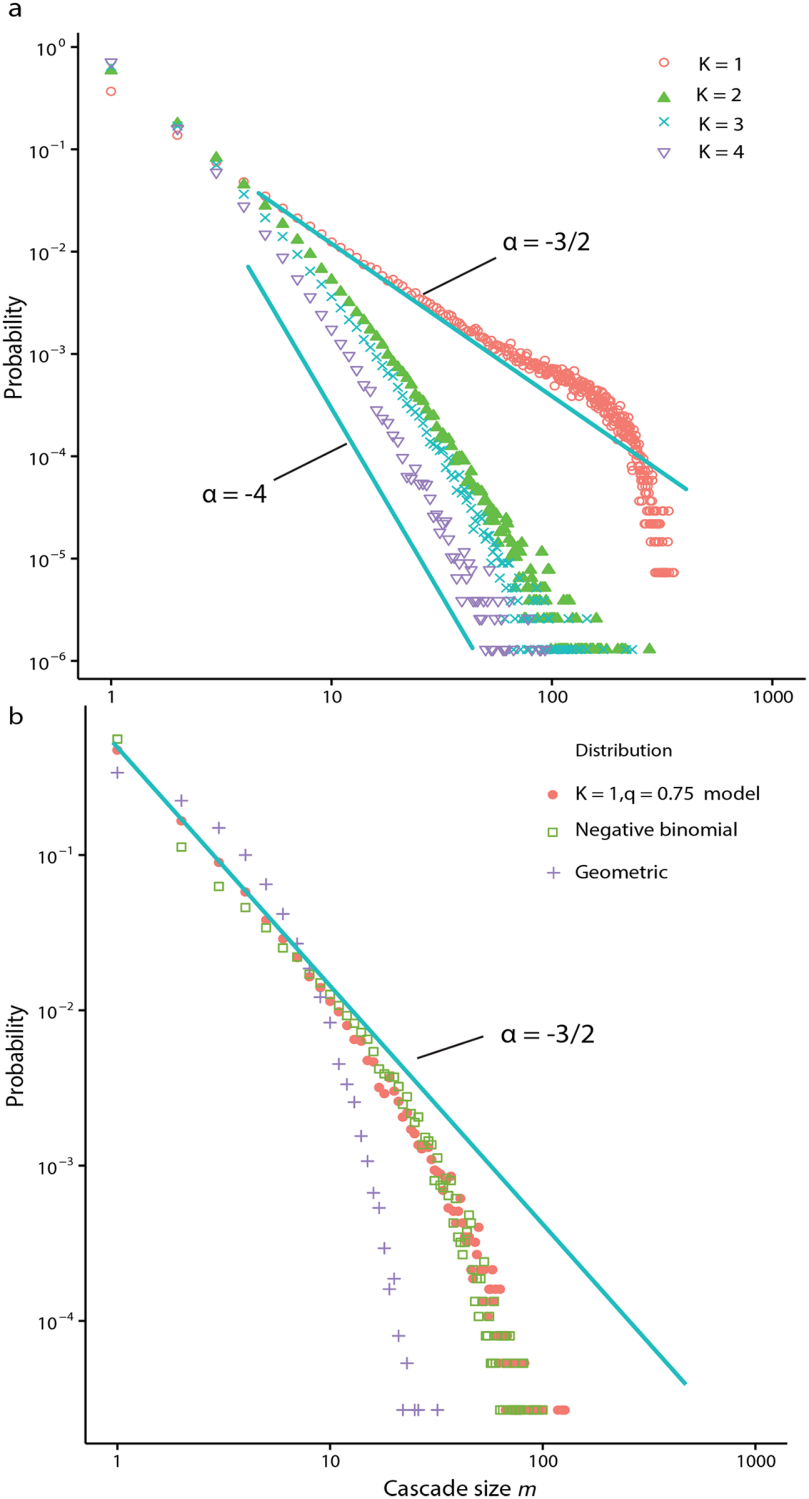
Log-log probability plots of cascade distributions. a) log-log probability plot of absolute cascade sizes when the system is near the transition value *q* = 1 for *K* = 1, 2, 3, 4 with *N* = 1000. The case *K* = 1, corresponding to the maximal coupling strength, displays an exponentially truncated tail due to finite size effects. b) log-log probability plot of cascade size for the system with parameters *K* = 1, *q* = 0.75 and *N* = 1000 (filled circles), compared to a geometric distribution (crosses) of equal mean, and a negative binomial (open squares) with both mean and variance matched.

The details of the derivations of Eqs (7) and (8) are presented in the methods section. When *q* = 1 the asymptotic cascade distribution takes the form of a power law with exponent −3/2, consistent with the infinite sub-critical Galton-Watson process [59, 60]. For *q* ≠ 1 Eq (8) represents a truncated power law. Fig 3a displays the distribution of absolute cascade sizes for various *K* near the critical point of *q* = 1, obtained via simulation, reflecting the findings for *K* = 1.

### Analysis and approximation of finite state-space models with *K* ≥ 1

When *K* > 1 each agent requires more than one pulse to induce it to a firing state, from the rest state. As a result, this dampens the ability of cascades to sweep through the entire system. Fig 3a displays the distributions of cascades sizes for *K* = 2, 3, 4 when *q* = 1. The exponents are estimated via maximum likelihood estimation (MLE) and the distribution fit tested using the Kolmogorov-Smirnov test. Estimates of the exponent (with standard error in parenthesis) range from *α* ≈ −2.25(0.001) for *K* = 2, to *α* ≈ −3.5(0.06) for *K* = 4, although the quality of the power law fit decays rapidly as *q* deviates from the critical value *q* = 1. We leave the derivation of a closed-form expression for the cascade distribution (equivalent to Eq (8)) when *K* > 1 for future research. Instead, the negative binomial distribution is sufficient for expressing the approximate moments of the cascade distribution in terms of *q* < 1.

#### Fitting a negative binomial distribution

**Fig 4:**
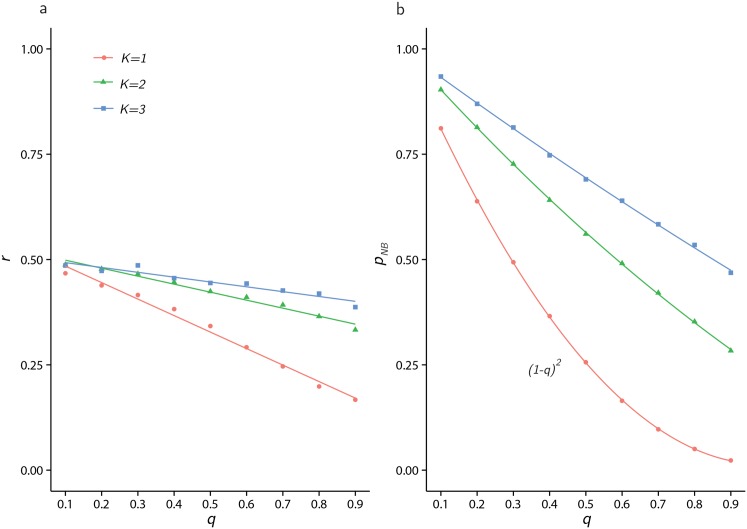
Extracting negative binomial parameters. When approximating the distribution of cascade sizes with a negative binomial distribution with parameters *r* and *p*_*NB*_, the parameters of moment matched negative binomial distributions are presented as a function of *q*, for *K* = 1, 2, 3. a) *r* and b) *p*_*NB*_.

Even though the mean and variance of the *K* = 1 cascade distribution can be expressed in closed form using special functions, we provide numerical evidence for a range of *K* values showing that a negative binomial distribution [61] may be used as a good approximation to the cascade distribution, when *q* < 1. Fig 3b shows the cascade distribution *K* = 1, *q* = 0.75 compared to a moment-matched negative binomial distribution with good agreement. Fig 4 shows how the parameters, *r* and *p*_*NB*_, of moment matched negative binomial distributions vary with *q*. Except for the case of *p*_*NB*_ when *K* = 1, both sets of parameters can be well approximated as varying linearly with *q*, for all *K* tested. The benefit of this approach is that the moments of the cascade distribution are easily expressed in terms of *q*, the key parameter of interest.

**Fig 5:**
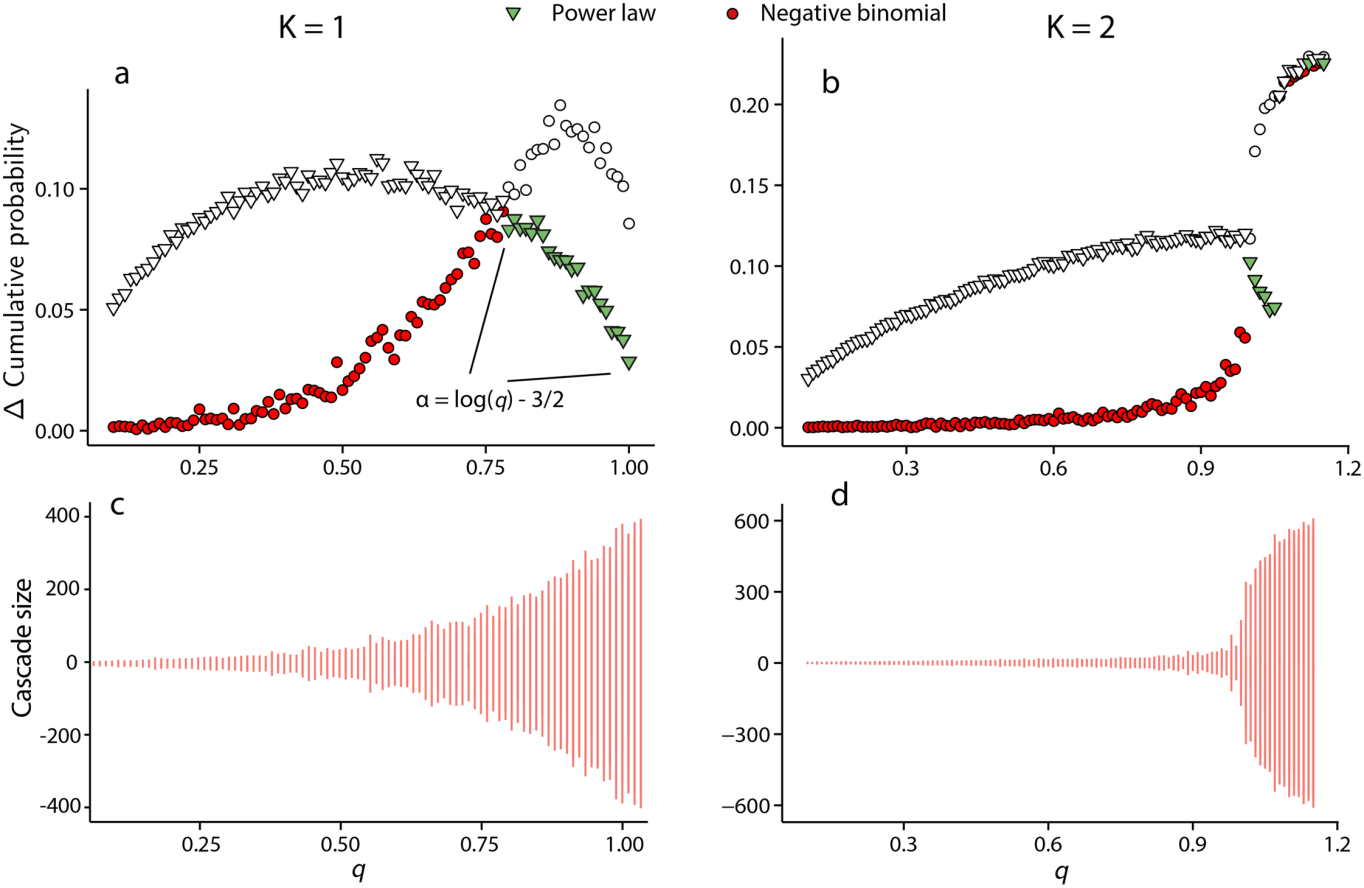
Kolmogorov-Smirnov statistic values obtained by fitting a power law and negative binomial model to absolute cascade size data. Filled shapes represent the minimum divergence of the two fits, calculated using the Kolmogorov-Smirnov statistic. *N* = 1000 for all panels. a) *K* = 1 and negative binomial fits well for *q* < 0.6, but power law (zeta distribution) is a better fit for *q* > 0.79, with exponent $\alpha =\log\left(q\right)-\frac{3}{2}$ in this region. b) *K* = 2 and negative binomial provides a good fit up to *q* < 0.85. c) and d) show the typical cascade sizes versus *q* for the case *K* = 1 and *K* = 2 respectively.

In Fig 5a and 5b, the Kolmogorov-Smirnov test statistic (see [62] for methodological details) is reported for both a power law and negative binomial fit, and the regions of *q* in which each distribution provides the best relative fit to the distribution of (*K*, *q*) is highlighted (via filled shapes). In the case *K* = 1, the negative binomial provides a good fit for 0 < *q* < 0.6, and the power law provides a better relative fit in the range 0.79 ≤ *q* ≤ 1.

Cascades occur with both positive and negative signs, with equal probability. As a result, we obtain the (approximate) full distribution of cascades sizes (both negative and positive) as a mixture distribution of two equally weighted negative binomial distributions, symmetric about 0. Using standard moment calculations (see methods) we obtain the variance of this full distribution written in terms of the parameters of the negative binomial distribution parameters, considered as a function of *q*

$$\begin{array}{c} \sigma^{2}\left(q\right)=\frac{1}{p_{NB}{\left(q\right)}^{2}}\left(r\left(q\right)\left(1-p_{NB}\left(q\right)\right)+{\left[p_{NB}\left(q\right)+r\left(q\right)\left(1-p_{NB}\left(q\right)\right)\right]}^{2}\right) \end{array}$$ (9)

where *r*(*q*) = *a*_1_ + *a*_2_ *q*, *p*_*NB*_(*q*) = *b*_1_ + *b*_2_ *q* + *b*_3_ *q*^2^ and the constants *a*_1_, *a*_2_, *b*_1_, *b*_2_, *b*_3_ vary with each value of *K* (see Fig 4). For *K* = 1, *p*_*NB*_(*q*) = (1 − *q*)^2^ and mean values of *a*_1_ and *a*_2_ over 1000 observations are 0.53(0.016) and −0.40(0.024), respectively (standard deviation displayed in parenthesis). For *K* = 2, *a*_1_ = 0.52(0.03), *a*_2_ = −0.19(0.04) and *b*_1_ = 0.96(0.007), *b*_2_ = −0.77(0.01), *b*_3_ = 0.

When *K* = 1, the standard deviation can be written

$$\begin{array}{c} \sigma \left(q\right)={\left[\frac{\left(a_{1}-a_{2}q\right)\left(1-{\left(1-q\right)}^{2}\right)}{{\left(1-q\right)}^{4}}+{\left(1+\frac{\left(a_{1}-a_{2}q\right)\left(1-{\left(1-q\right)}^{2}\right)}{{\left(1-q\right)}^{2}}\right)}^{2}\right]}^{1/2}. \end{array}$$ (10)

A similar calculation can be performed for excess kurtosis, and is presented in the methods section.

### Financial market model

As an application of the (*K*, *q*) process, we illustrate how it may be incorporated into a simple model of financial returns. Let the logarithmic price return, *r*_*t*, Δ*t*_ over some interval Δ*t* starting at time *t* be given by

$$\begin{array}{c} r_{t,\Delta t}=\log P_{t+\Delta t}-\log P_{t}=\log\left(\frac{P_{t+\Delta t}}{P_{t}}\right) \end{array}$$ (11)

where, *P*_*t*_ is the price of a traded asset—such as a stock, bond or commodity. We regard the cascade sizes *m*, generated by the actions of traders in our model, as excess demand for a financial asset. When the excess demand is positive, the price of the asset will increase and vice-versa it will decline when excess demand is negative (excess supply). Given an excess demand (cascade size) of *m*, the price impact function [63], *f*, dictates the magnitude of the price change by mapping *m* to a positive real variable, so that f(m)∈R. In order to keep the model as simple as possible, we follow previous works [7] and take *f*(*m*) = *λm*, for some *λ* > 0 referred to as the market depth parameter. For a description of more realistic choices of price impact function, see the article by R. Almgren [64].

To summarise, by rearranging Eq (11) and setting Δ*t* = 1, we can formulate the 1-period price update

$$\begin{array}{c} P_{t+1}=P_{t}e^{\lambda m} \end{array}$$ (12)

where we have identified *λm* with the 1-period return: *r*_*t*, 1_. More generally, let *M* be a variable representing observations {*m*_1_, *m*_2_, …} from the (*K*, *q*) cascade process. Then we can write the *n*-period price as

$$\begin{array}{c} P_{n}=P_{0}e^{\lambda \sum_{i=1}^{n}m_{i}}. \end{array}$$ (13)

Recall that for the case *K* = 1, cascades are statistically independent identically distributed events, and trades occur in continuous time with an exponentially distributed inter-arrival times. In order to fully specify the price process, we write this as a compound Poisson process

$$\begin{array}{c} J\left(t\right)=\sum_{i=1}^{n(t)}M_{i}. \end{array}$$ (14)

Each *M*_*i*_ follows the distribution of *M* and {*n*(*t*)} is a Poisson process with rate *θ*, used to describe the time between trades (and any ensuing cascades). Finally, for time *t* > 0 we write,

$$\begin{array}{c} P_{t}=P_{0}e^{\lambda J\left(t\right)}\Rightarrow r_{0,t}=\lambda J\left(t\right). \end{array}$$ (15)

Using standard results of compound Poisson processes, and noting that the mean cascades size is zero due to symmetry, the variance of *J*(*t*) can be given as: $\text{Var}\left(J\left(t\right)\right)=\theta t\;E\left\{M^{2}\right\}$. When *M* is approximated as a mixture distribution of two equally weighted negative binomial distributions symmetric about 0 we have

$$\begin{array}{c} \text{Var}\left(J\left(t\right)\right)=\theta t\sigma^{2}\left(q\right) \end{array}$$ (16)

where *σ*^2^(*q*) is given by Eq (9). This connects the variance of model price returns, of all periods, to the network coupling probability.

**Fig 6:**
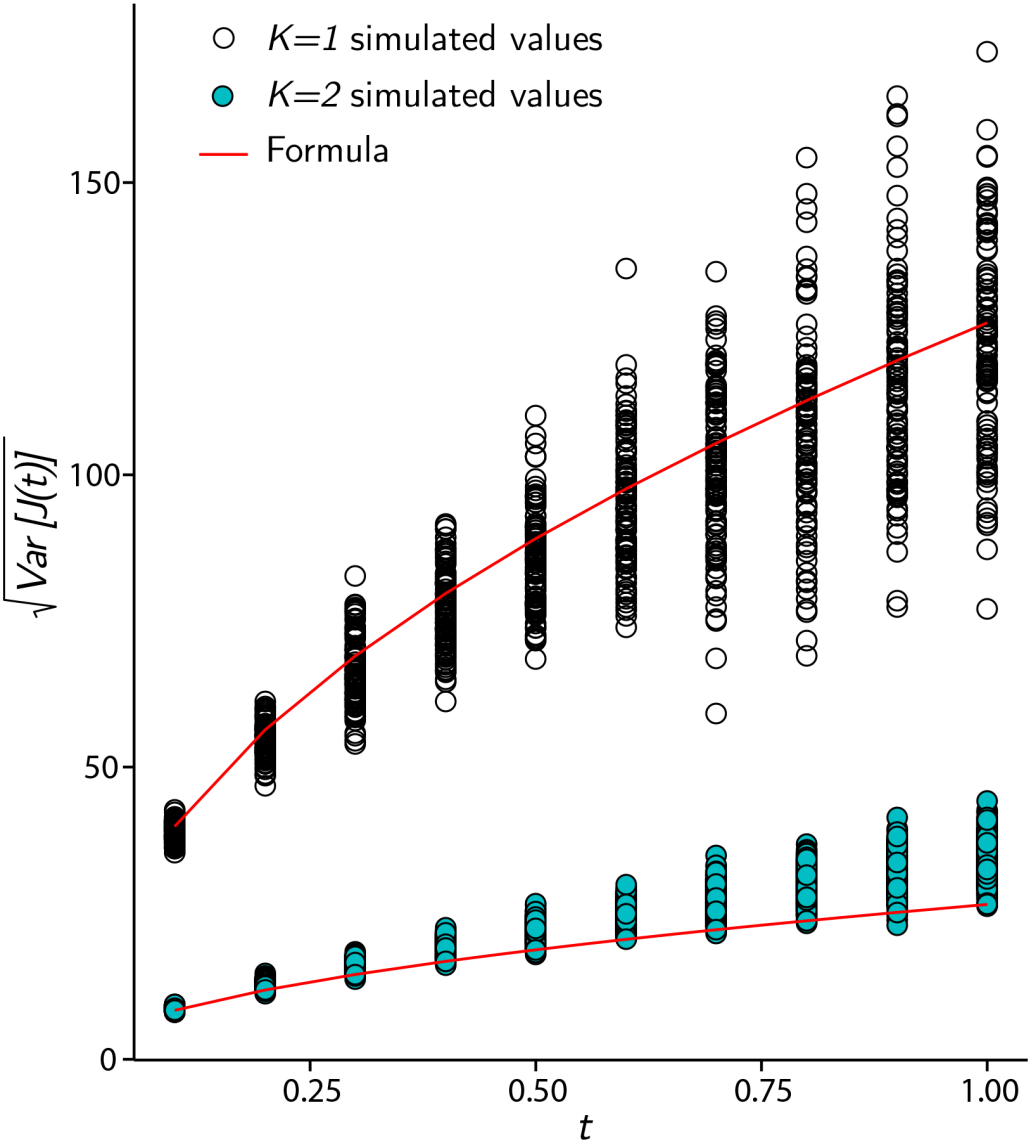
Comparison of simulated standard deviation and closed form approximation. Simulated values of the standard deviation (volatility) of period *t* returns, for 0 < *t* ≤ 1, given by Eq (15) with *λ* = 1, compared to values given by Eq (9). Values for both *K* = 1 (open circles) and *K* = 2 (filled circles) are displayed. For *K* = 2, the formula underestimates the mean simulated value, due to dependence between returns and deviation from the compound Poisson process.

A comparison between simulated values of $\sqrt{\text{Var}(J(t))}$ (the standard deviation of period *t* returns *r*_0,*t*_ with *λ* = 1) and $\sqrt{\theta t}\sigma \left(q\right)$, using Eq (9), is shown in Fig 6. Parameter values used are *N* = 1000 and *q* = 0.6. For each *t* shown, 100 values of $\sqrt{\text{Var}(J(t))}$ are plotted, where the variance is taken over 200 period *t* returns. By appealing to standard results concerning random diffusion without drift between two symmetric absorbing barriers [65], *θ* = *N*/*K*^2^.

### Volatility clustering and semi-infinite state-space model

While the finite state-space model describes a plausible trader interaction model, in its current form (without a time-varying network coupling probability parameter *q*) it does not account for certain features observed in the volatility autocorrelation of real asset returns. In particular, the finite state-space model does not exhibit volatility clustering, identified by a slow decay of return volatility autocorrelation, which is a well-documented property of asset returns [32, 66] that manifests over different times-scales and assets classes. Asset return volatility autocorrelation, *C*, is measured in a direct way by computing the autocorrelation between lagged observations of log-returns

$$\begin{array}{c} C\left(L,d\right)=\text{Corr}\left(|r_{t+L,d}\left|,\right|r_{t,d}|\right), \end{array}$$ (17)

where the log-return *r* is defined by Eq (11), and *d* represents the horizon over which the return is computed.

In this section, the finite state-space model is adapted to allow for the integrate phase of agents—representing the accumulation of agents’ information or sentiment—to take place over a semi-infinite state-space. For this model, in addition to zero serial correlation of log-returns, numerical results show volatility clustering is produced generically, and a condition under which volatility autocorrelation may exhibit a long-memory pattern [30] is described. Although such a model may not be representative of high-frequency trading, the accumulation of information represented as transitions on an unbounded set with a single firing state is congruent with the behaviour of agents with heavy-tailed inter-trade durations. The decision making of traders whom employ buy-and-hold strategies, or who temporarily withdraw from the market or who otherwise trade infrequently [67], may be better described by the semi-infinite state-space model, rather than the finite state-space model.

The semi-infinite state-space model is obtained by permitting agents to possess a single firing state, and allowing state transitions to take place between states labelled with the integers {… , − 2, − 1, 0, … , *K* − 2, *K* − 1}, with the firing state represented by the state labelled as *K* and the reset state labelled 0 (see methods section). While the mean first passage time for an unbiased nearest-neighbour random walk of a single particle in a semi-infinite region is well-known be infinite [65], because pulse-coupling between *N* agents induces agents to move closer to the firing state, rather than away from it, each agent can be considered to undergo a biased random walk in the semi-infinite region. Therefore, when there is a non-zero pulse-coupling probability *p* the mean first passage time, $\tau (\hat{p},K)$, for an agent to reach the firing state is finite, and given by

$$\begin{array}{c} \tau \left(\hat{p},K\right)=\frac{x_{0}+K}{2\hat{p}-1},\;\;\frac{1}{2}<\hat{p}\leq 1,\;\;K>0, \end{array}$$ (18)

where $\hat{p}$ is the biased probability, starting at position *x*_0_, of moving towards the firing state *K* at each time step.

Two distinct scenarios of the financial market model, given by Eqs (11)–(13), are numerically analysed. The first consists of agents with homogeneous firing thresholds (all agents possess the same threshold, *K* > 0). For the second case, agents in the population possess differing firing thresholds, described by a probability distribution *ϕ*(*K*) over the *N* agents. In this case two probability distributions, given by *ϕ*, are examined. For the first case,

$$\begin{array}{c} \phi \left(K\right)∼{\left(1+K_{\text{max}}-K\right)}^{-\alpha }, \end{array}$$ (19)

with parameters *α* > 1 and *K* ∈ [1, *K*_max_], and is taken to reflect a market that is composed of a small number of relatively influential traders (small *K*), together with a larger number of easily influenced traders (large *K*). The second probability distribution examined is,

$$\begin{array}{c} \phi \left(K\right)∼{\left(1-p_{G}\right)}^{K-1}p_{G}, \end{array}$$ (20)

with parameter 0 < *p*_*G*_ ≤ 1 and *K* ≥ 1, which describes a geometric distribution.

**Fig 7:**
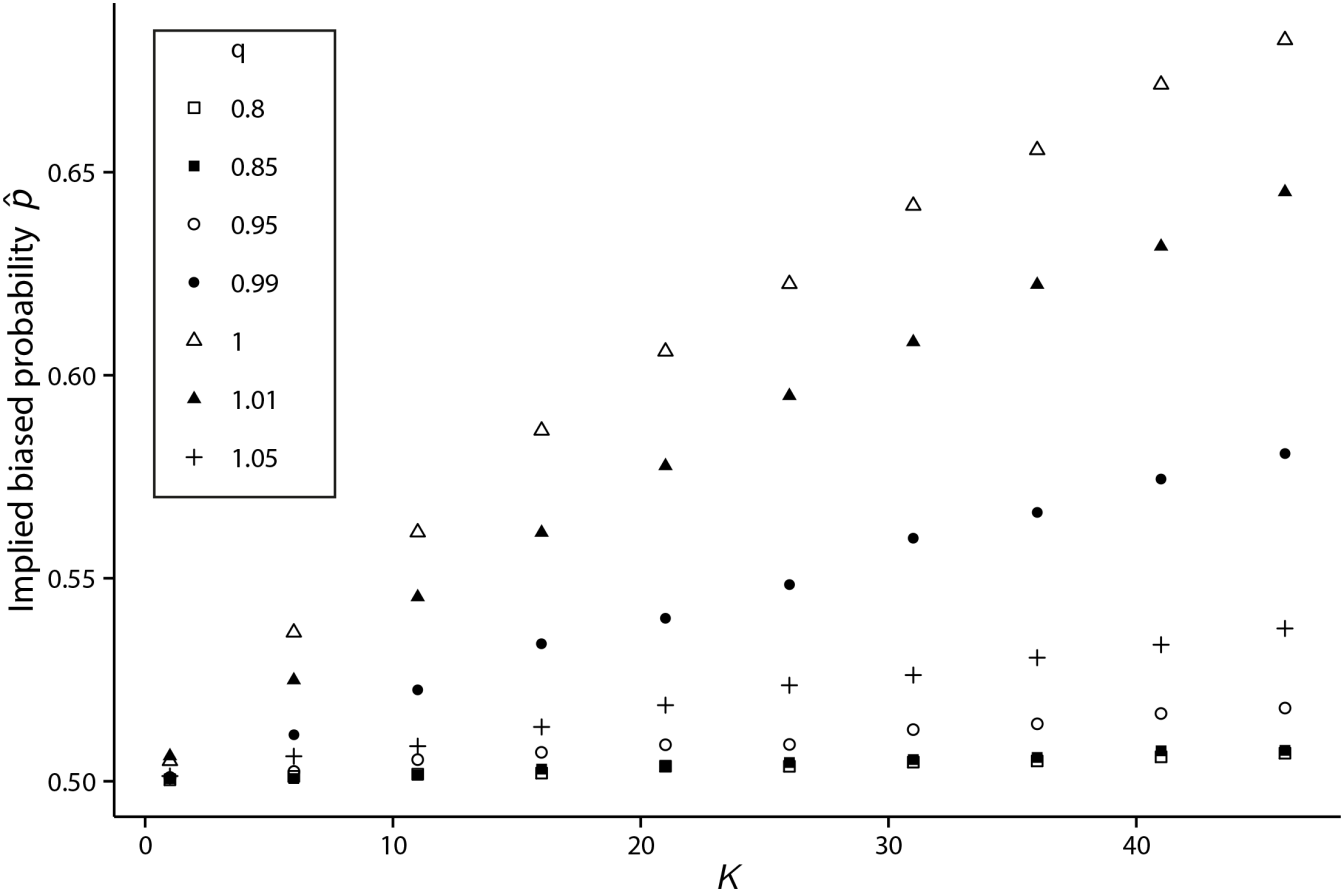
Implied biased probability $\hat{p}$ versus threshold *K* for a range of values for *q*. For a range of *q* values, representing the pulse-coupling probability parameter, from *q* = 0.8 to *q* = 1.05, a random agent from a population of agents each with identical threshold *K*, is selected and its mean hitting time to the threshold is computed from 10,000 simulations. Using Eq (18), the implied bias probability, $\hat{p}$, is then computed. For every *K* value tested, $\hat{p}$ attains its maximum value when *q* = 1.

For a homogeneous population of agents each having an identical firing threshold *K* > 0, it is noted $\hat{p}$ defined by Eq (18) varies non-linearly with the pulse-coupling probability *p* = *qK*/*N* as can be seen in Fig 7.

It is clear that when *q* → 0, $\hat{p}\to 0.5$, since $\tau (\hat{p},K)\to \infty$ in this case. As *q* surpasses the critical value of *q* = 1, a randomly identified agent becomes more likely to reach the firing threshold as part of a large cascade, making fewer agents available from which it may receive pulse-coupling events upon reset, on average. As a result, this agent’s random walk in the semi-infinite state-space when *q* ≥ 1 is less biased by pulse-coupling effects when compared to the case *q* = 1.

**Fig 8:**
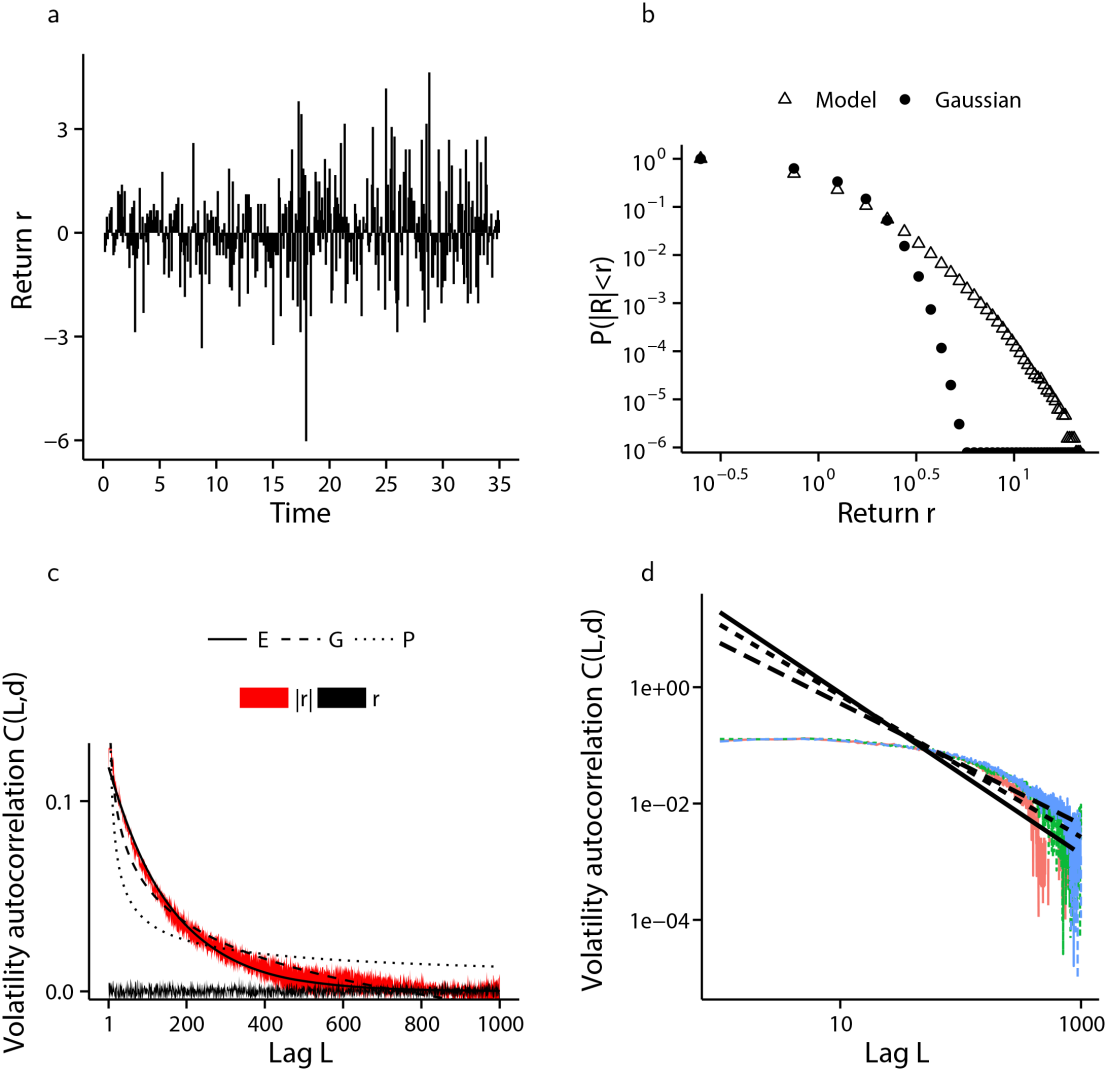
Simulation of the financial market model with semi-infinite state-space and homogeneous firing thresholds. The results from ten independent simulations each of 150,000 cascades is presented. (a) A sample from one of the ten simulated log-returns series. (b) Comparison of the distribution of log-returns arising from the model (△) with a moment matched Gaussian distribution (•), shown in log-log scale clearly showing fat-tails. (c) one standard deviation envelope around the mean volatility autocorrelation of log-returns (*r* and black) and absolute log-returns (|*r*| and red) with lag *L*, together with the non-linear least squares fit of exponential (labelled E—solid line), logarithmic (labelled G—dashed line) and hyperbolic (labelled P—dotted line) decay functions, with exponential decay providing the best fit. (d) a random sample of three out of ten volatility autocorrelation computations with hyperbolic decay lines of best fit, shown in log-log scale.

**Fig 9:**
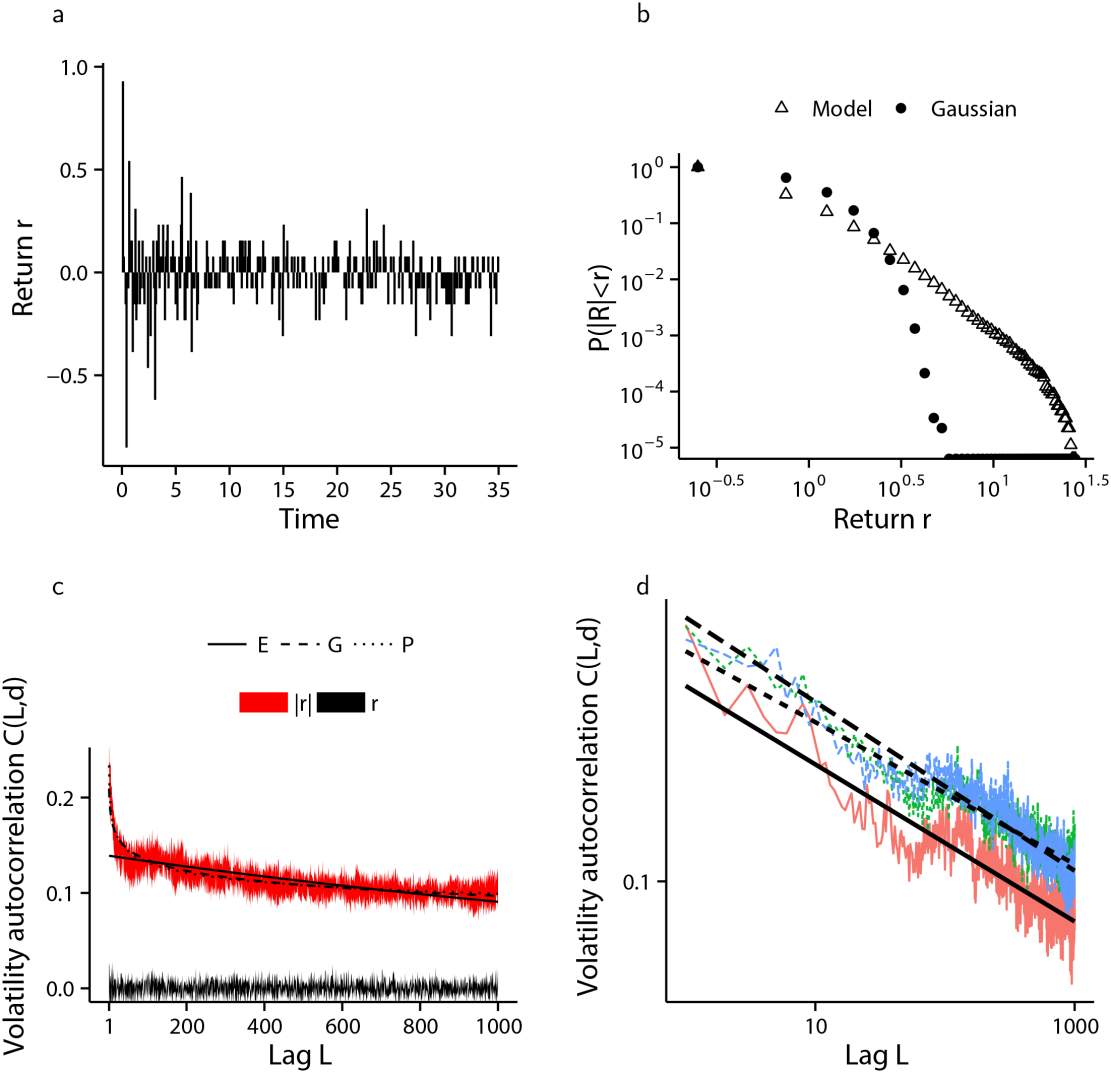
Simulation of the financial market model with semi-infinite state-space and inhomogeneous firing thresholds. The results from ten independent simulations each of 150,000 cascades is presented where firing thresholds are distributed among the agent population according to Eq (19). (a) A sample from one of the ten simulated log-returns series. (b) Comparison of the distribution of log-returns arising from the model (△) with a moment matched Gaussian distribution (•), shown in log-log scale clearly showing fat-tails. All simulated data used. (c) one standard deviation envelope around the mean volatility autocorrelation of log-returns (*r* and black) and absolute log-returns (|*r*| and red) with lag *L*, together with the non-linear least squares fit of exponential (labelled E—solid line), logarithmic (labelled G—dashed line) and hyperbolic (labelled P—dotted line) decay functions, with hyperbolic decay providing the best fit. All simulated data used. (d) a random sample of three out of ten volatility autocorrelation computations with hyperbolic decay lines of best fit, shown in log-log scale.

Fig 8, presents the results of the numerical simulation of the financial market model when agents have an identical firing threshold of *K* = 2, while Fig 9 shows the same information when firing thresholds are distributed amongst the agent population according to Eq (19), with parameter values *K*_max_ = 20 and *α* = 1.93.

**Fig 10:**
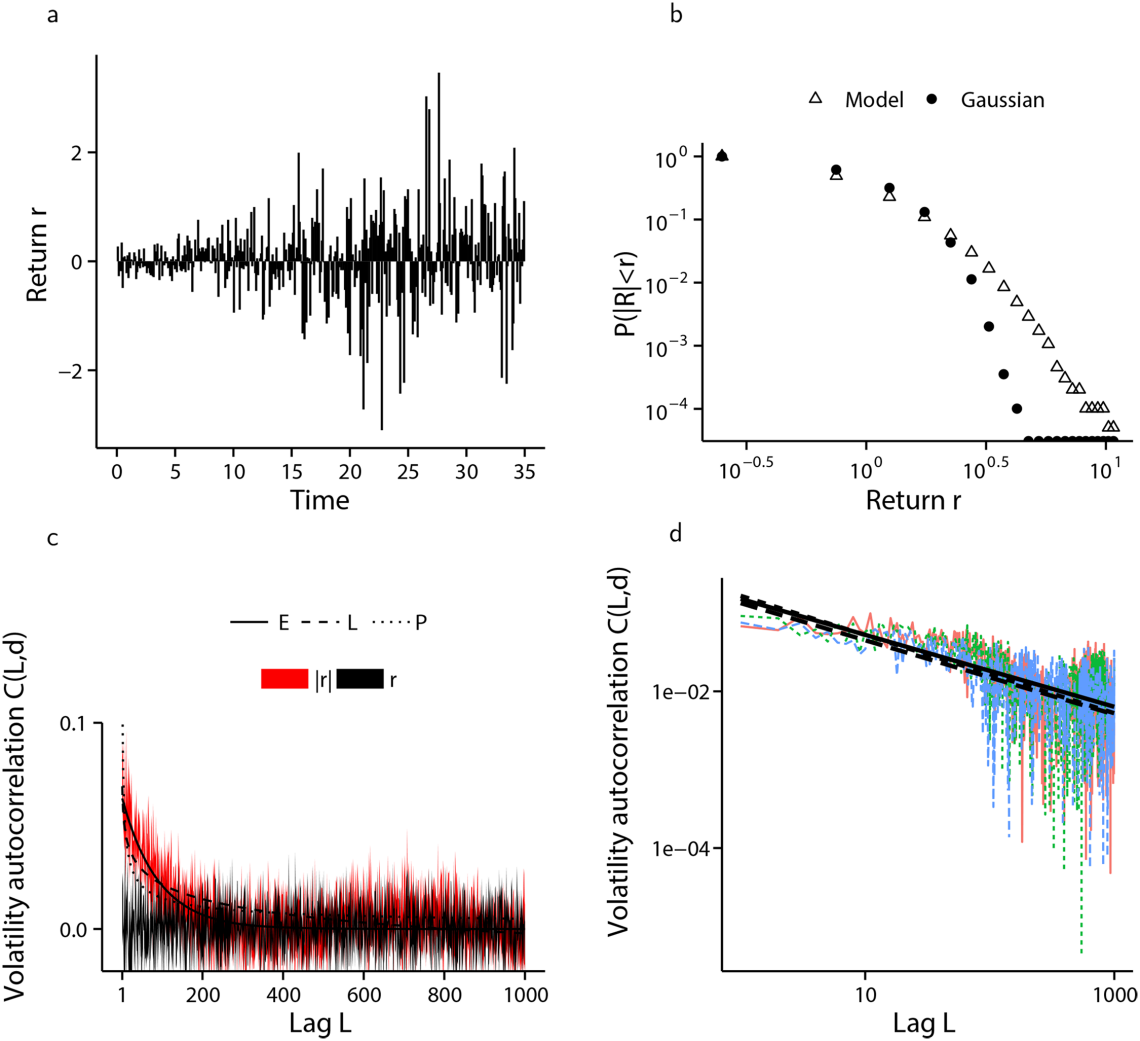
Simulation of the financial market model with semi-infinite state-space and inhomogeneous firing thresholds. The results from ten independent simulations each of 150,000 cascades is presented where firing thresholds are distributed among the agent population according to a geometric distribution, given by Eq (20), with parameter $\frac{1}{6}$. This distribution is right-skewed meaning there are many more agents with low *K* firing thresholds than agents with high *K* thresholds. In contrast to when the distribution of firing thresholds is left-skewed, as in Eq (19), hyperbolic decay is not exhibited. (a) A sample from one of the ten simulated log-returns series. (b) Comparison of the distribution of log-returns arising from the model (△) with a moment matched Gaussian distribution (•), shown in log-log scale clearly showing fat-tails. All simulated data used. (c) one standard deviation envelope around the mean volatility autocorrelation of log-returns (*r* and black) and absolute log-returns (|*r*| and red) with lag *L*, together with the non-linear least squares fit of exponential (labelled E—solid line), logarithmic (labelled G—dashed line) and hyperbolic (labelled P—dotted line) decay functions, with exponential decay providing the best fit. All simulated data used. (d) a random sample of three out of ten volatility autocorrelation computations with hyperbolic decay lines of best fit, shown in log-log scale.

Volatility clustering is generated which decays exponentially in the homogeneous case, and hyperbolically when agent pulse-coupling thresholds are inhomogeneous and distributed according to Eq (19). The hyperbolic decay visible in Fig 9c is exhibited for all *α* tested in the range *α* = 1.5 to *α* = 5, and *K*_max_ ∈ [10, 100], although the hyperbolic nature of the decay becomes less pronounced as the distribution *ϕ* deviates from the power-law form given by Eq (19), and becomes virtually non-existent when *ϕ* is changed so as to produce a market consisting of many relatively influential (low *K*) agents together with fewer easily influenced (large *K*) agents. Fig 10 demonstrates this change when the distribution of agent firing thresholds is geometric, according to Eq (20), with parameter $p_{G}=\frac{1}{6}$.

In terms of economic implications, these results are consistent with previous studies that incorporate heterogeneity of agent time-scales into statistical models of market volatility [68–70], although in the models presented here, an explicit trader interaction mechanism is responsible for patterns in asset volatility autocorrelation. Moreover, this model shows how hyperbolic decay of volatility autocorrelation, associated with statistical long-memory, may be the result of a leadership effect [71] resulting from the structure and composition of markets with agents of differing trading, or informational, thresholds. Such an understanding may aid investors in determining appropriate trading strategies for a given market, or in examining if a particular trade or market is crowded [72], with an abundance of either influential, or easily influenced, traders.

### Comparison to market data

#### Equity returns

**Fig 11:**
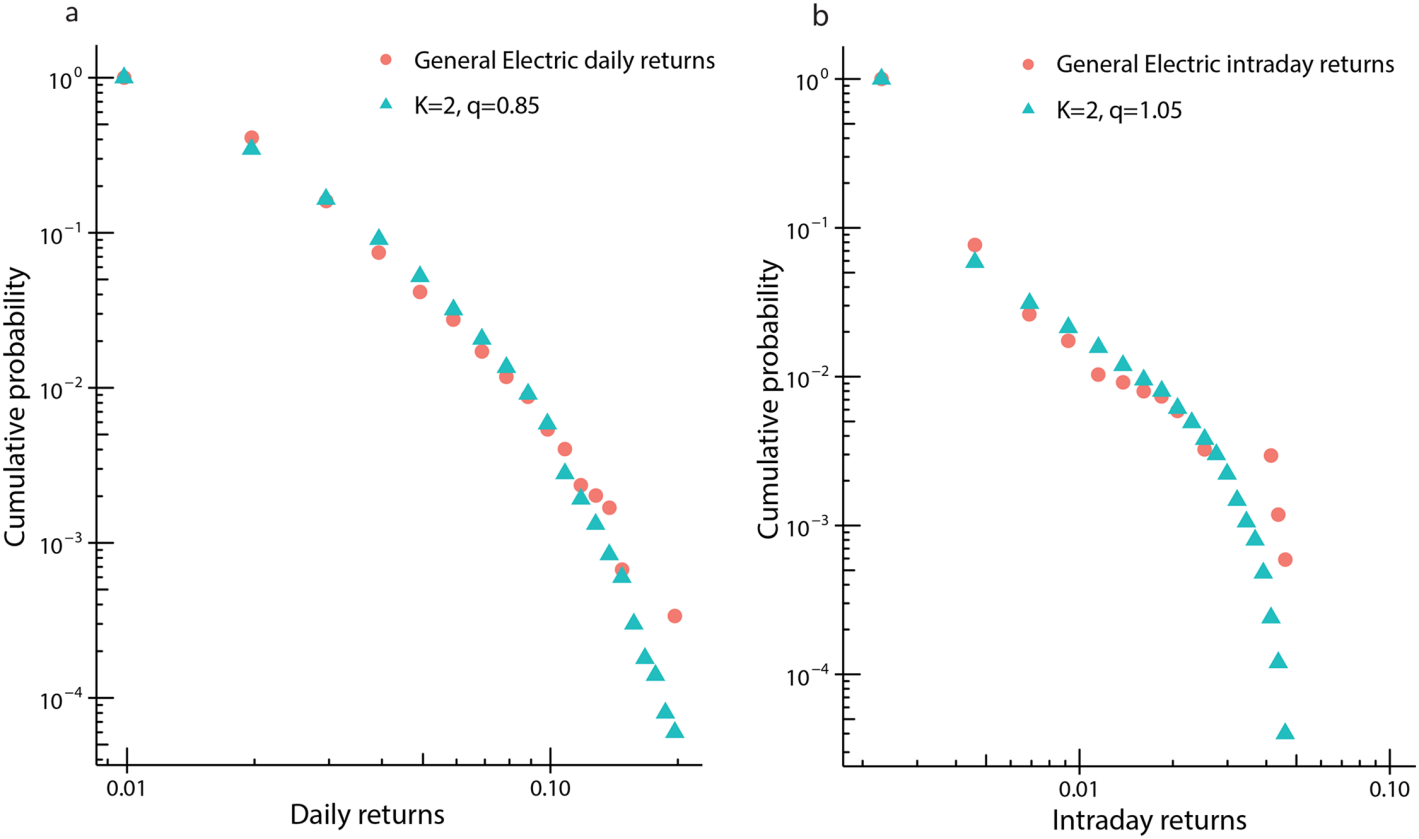
log-log probability plots of (*K*, *q*) compared to mid price market data. a) The cumulative probability distribution of daily non-zero returns for a randomly selected stock, General Electric, computed using data for the period January 3 2003 to Feb 6 2015 (3045 points) (filled circles). Overlaid is the distribution of *K* = 2, *q* = 0.85 using market depth *λ* = 8.2 × 10^−3^. b) The cumulative probability plot of the same stock as in a), but using intraday price returns computed at, on average, 1.5 second intervals over the period May 6 2010 (flash crash), 14:05 to 15:25 (3390 data points) (filled circles). Overlaid is the distribution of *K* = 2, *q* = 1.05 using market depth *λ* = 5.2 × 10^−5^. In both cases *N* = 1000 is used.

As an example of the use of the (*K*, *q*) finite state-space financial market model, we compute indicative values of *K* and *q* (with *N* = 1000 nominally set) to estimate the distribution of market returns for a randomly selected instrument (General Electric equity stock) over two different time scales. The finite state-space model is used here, rather than the infinite state-space model, as the distribution of log-returns is not significantly altered by volatility clustering. Our results are summarised in Fig 11.

To produce the plot shown in Fig 11a, we take end of day closing prices from January 3 2003 to February 6 2015, compute the daily log-return distribution and compare this to a *K* = 2 *q* = 0.85 distribution with a market depth parameter, *λ*, of 8.2 × 10^−3^. For Fig 11b we use intraday data to compute non-zero log-returns, of approximately 1.5-second intervals, over a period of time capturing the so-called flash-crash of May 6 2010. In particular, we use data from May 6 2010 14:05 to 15:25 (EST), resulting in 3390 data points to compute the cumulative probability and compare this to a *K* = 2, *q* = 1.05 distribution with *λ* = 5.2 × 10^−5^. While these comparisons are provided as illustrative, rather than representing detailed statistical best-fits, it is of interest to note Fig 11b showing *q* > 1 during the extremely volatile period of the flash-crash, as one might expect.

#### Option on an equity index

One of the reasons for the persistence of Gaussian-based models of financial returns, is the body of knowledge accumulated to price derivative contracts [53]—and most notably the framework of Black, Scholes and Merton (BSM) [51], that enables a price of certain derivative contracts to be computed using closed form formulae. To account for the gap between real market characteristics and the Gaussian assumptions that underpin the BSM framework, traders make an adjustment to the volatility of returns (a parameter of the BSM pricing formula) to account for the observed heavy tails of financial returns [52, 73]. As a result, when the volatility used to price derivative contracts is plotted against the strike price of option contracts, the resulting implied volatility curve is known as the volatility smile, due to its curved appearance, indicating larger values at the extremes of strike price.

**Fig 12:**
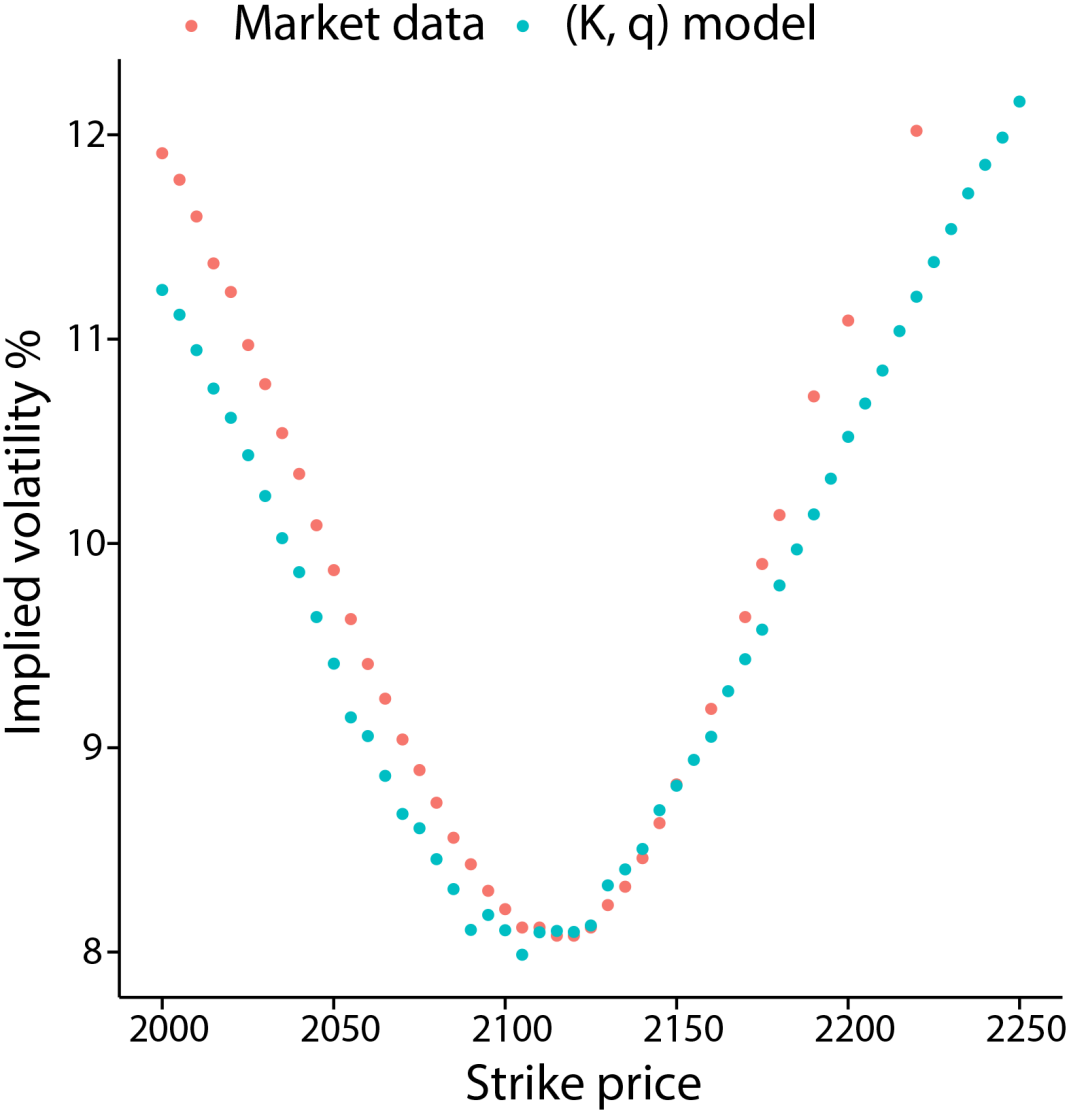
Fitting the implied volatility smile for index options. The Black-Scholes implied volatility smile obtained from market data of European call options on the SPXpm index is compared to the implied volatility obtained from empirical option prices, generated using a *K* = 2, *q* = 0.78 model.

We demonstrate that the (*K*, *q*) model is able to recover approximate market prices of European options (see methods section) by matching the market price implied volatility smile. We use data consisting of European call options written on the afternoon settled S&P 500 (SPXpm) index as of November 25 2014, with an expiry of December 20 2014. We use options with a strike price between 2000 to 2250, with the SPXpm index level at 2067.03 at the close of November 25 2014. Fig 12 demonstrates the recovered volatility smile for these data.

The fit, while not perfect, does match the general shape of the smile well. To obtain the volatility smile, we take a large number of draws from a (*K*, *q*) process, and apply the empirical option pricing procedure outlined in [74] to obtain prices for call options of given expiry and strike prices. The implied volatility is then recovered by using a simple numerical root-search. The recovered implied volatilities are compared to those obtained via the market data, and the process is repeated with different values of *K* and *q* until a suitable fit is found.

## Methods

### Model description

#### Finite state-space

The model consists of *N* agents, or traders, operating in a financial market for a single asset, and represented as integrate-and-fire stochastic pulse-coupled oscillators connected via an all-to-all interaction network with *N* vertices. Let the information state of agent *i* be given by *θ*_*i*_(*t*). In the case where all agents possess identical information thresholds determined by an integer *K* > 0, each agent can transition stochastically between 2*K* + 1 information states enumerated as {0, 1, 2, …, 2*K*}. Inbetween trades (that is, during the integrate phase of the oscillators), each agent accumulates private information unobserved by other agents at times described by a continuous exponential random variable with mean 1. In the absence of any structure relating to how agents accumulate such private information, this is represented by the agents randomly transitioning between the information states of the system (similar to so-called noise traders), thus agents perform a random-walk with step size equal to one over the finite set of integers {0, 1, …, 2*K*}. When an agent has accumulated enough information so as to reach either state 0 or 2*K* (the firing states), a market transaction is executed that reduces or increases (with equaly probability) the market asset price, respectively. Each transaction is assumed to impact the market price of the traded asset according to some specified price-impact function [63]. Since market prices are observed by all agents, for each agent that transitions to the firing state *X*, where *X* = 0 or *X* = 2*K*, each market agent not already in one of the firing states updates their information set by moving one state closer to state *X*, independently with probability equal to *p*, parametrised as *p* = *Kq*/*N*, with *q* being a parameter such that 0 < *q* < *N*/*K*. With probability (1 − *p*), an agent ignores the change in the asset price and does not update their information set. As a result, a cascade may form with agents inducing other agents into the same firing state who are then assumed to mimic the same buy or sell decision as the agent that instigated the cascade. The cascade size, *m*, is defined by the number of agents, *m*_*A*_, that accumulate in the firing state during a cascade, prior to being reset to state *K*. If the trade instigated by the originator of the cascade is a buy, we take *m* = |*m*_*A*_| otherwise we take *m* = −|*m*_*A*_|. It is assumed that cascades form instantaneously. After a reset, all agents resume stochastic accumulation of private information until the next transition into a firing state occurs, and the system repeats. The cascade process (*K*, *q*) is taken to refer to the sequence of cascade sizes, {*m*_1_, *m*_2_, …}, generated from such a system.

#### Semi-infinite state-space

The semi-infinite state-space model consists of agents with a single information threshold set at *K*, which may differ amongst agents. Let *K*_*u*_ represent the information threshold of an agent receiving a pulse-coupling event. As a result, during the integrate phase, an agent with firing state *K* transitions between states enumerated by the infinite set of integers {… , − 2, − 1, 0, 1, … , *K* − 1, *K*}. Succinctly, the semi-infinite information state-space is simply the set of integers less than or equal to *K*. Upon an agent transitioning to the firing state, *K*, all other agents are either successfully pulse-coupled with probability *K*_*u*_ *q*/*N*, and transition closer to their firing state, or with probability 1 − *K*_*u*_ *q*/*N* the pulse-coupling in unsuccessful. Cascades are formed using the same mechanism described for the finite state-space case. Once a cascade has terminated, agents that were induced to trade are reset to state 0, and the system repeats as in the finite state-space case.

### Combinatorial methods for cascade probability

The composition [75] of an integer, *x*, is the sequence of strictly positive summands of *x*. That is, if *x* = *x*_1_ + *x*_2_ + … + *x*_*k*_ then the sequence {*x*_1_, *x*_2_, …, *x*_*k*_} is called a composition of *x*. There are exactly 2^*x* − 1^ distinct compositions of an integer *x*. We use the concept of integer compositions to derive Eqs (7) and (8). To make it clear when we are working with compositions we use the notation *x* = [*x*_1_, …, *x*_*k*_].

Consistent with Eqs (1) to (3), an arbitrary (unsigned) cascade of size *m* > 0, initiated by a single agent, may be written in composition form as *m* = [1, *x*_1_, …, *x*_*k*_], and therefore *m* − 1 = [*x*_1_, …, *x*_*k*_]. We identify *x*_*i*_ as being the number of internal nodes at level *i* in the tree representation of a cascade (see Fig 2).

We proceed by enumerating the ways such a cascade can arise. Given a cascade expressed as [*x*_1_, …, *x*_*k*_], at an arbitrary level *i* we have *x*_*i* − 1_ copies of a single level (*N* − 1 − *x*_1_ − … − *x*_*i*_)-ary tree. We then have

xi-1xiN-1-∑j=1i-1xjxi (21)

ways to select the *x*_*i*_ nodes. Proceeding recursively, we form the product

N-1x1...xi-1xiN-1-∑j=1i-1xjxi...xk-1xkN-1-∑j=1k-1xjxk=(N-1)!(N-m)!x1x2x2x3...xk-1xkx1!x2!...xk!, (22)

where the right hand side of the equality is achieved after pairwise cancellation and using the fact that *m* = 1 + [*x*_1_, …, *x*_*k*_]. Hence for a given composition (with *k* parts) we can write the probability as

$$\begin{array}{c} P\left(m\;|\;\left[x_{1},...,x_{k}\right]\right)=\frac{(N-1)!}{(N-m)!}\frac{x_{1}^{x_{2}}x_{2}^{x_{3}}...x_{k-1}^{x_{k}}}{x_{1}!x_{2}!...x_{k}!}p^{m-1}{\left(1-p\right)}^{Q}, \end{array}$$ (23)

where *p* is the probability that an agent is induced to a firing state during a cascade and *Q* is the perimeter of the tree representation of the cascade. By simple counting, and using the fact that $\sum_{i\geq 1,j>i}x_{i}x_{j}=\frac{1}{2}({\left(m-1\right)}^{2}-\sum_{i\geq i}x_{i}^{2})$ we can express $Q=m\left(N-m\right)+\frac{1}{2}{\left(m-1\right)}^{2}-\frac{1}{2}\sum_{i\geq 1}x_{i}^{2}$. By removing the composition-dependent term, $\frac{1}{2}\sum_{i\geq 1}x_{i}^{2}$, from *Q* since it is relatively small, we can write the unconditional probability of a cascade of size *m* as

$$\begin{array}{c} P\left(m\right)=\sum_{k\geq 1,[x_{1},..,x_{k}]}P\left(m\;|\;\left[x_{1},..,x_{k}\right]\right)\\ =p^{m-1}{\left(1-p\right)}^{m\left(N-m\right)+\frac{1}{2}{\left(m-1\right)}^{2}}\frac{(N-1)!}{(N-m)!}\sum_{k\geq 1,[x_{1},...,x_{k}]}\frac{x_{1}^{x_{2}}x_{2}^{x_{3}}...x_{k-1}^{x_{k}}}{x_{1}!x_{2}!...x_{k}!}\\ =p^{m-1}{\left(1-p\right)}^{m\left(N-m\right)+\frac{1}{2}{\left(m-1\right)}^{2}}\frac{(N-1)!}{(N-m)!}\frac{m^{m-2}}{(m-1)!}. \end{array}$$ (24)

For large *N*, *m*

(N-1)!(N-m)!=N-1m-1(m-1)!∼Nm-1 (25)

and

$$\begin{array}{c} \frac{m^{m-2}}{(m-1)!}=\frac{m^{m-1}}{m!}∼{\left(2\pi \right)}^{-\frac{1}{2}}m^{-\frac{3}{2}}e^{m} \end{array}$$ (26)

follow from Stirling’s approximation. The final equality in Eq (24) can now be rewritten as

$$\begin{array}{c} P\left(m\right)={\left(2\pi \right)}^{-\frac{1}{2}}{\left(pN\right)}^{m-1}{\left(1-p\right)}^{m\left(N-m\right)+\frac{1}{2}{\left(m-1\right)}^{2}}m^{-\frac{3}{2}}e^{m}, \end{array}$$ (27)

and recalling *p* = *qK*/*N*, with *K* = 1 as *N* → ∞ we obtain the asymptotic relation given by Eq (8).

### Mixture distribution moments and negative binomial denisty

First note that since cascade evolution is stochastic, each agent will undergo a random number of unsuccessful attempts before they are induced to fire—if at all. It is this simple observation that motivates the choice of the negative binomial statistical model to approximate the cascade distribution. The density of the negative binomial distribution used is

$$\begin{array}{c} \frac{\Gamma (x+r)}{x!\Gamma (r)}p_{NB}^{r}{\left(1-p_{NB}\right)}^{x},\;x=0,1,...,\;r>0,\;0<p_{NB}\leq 1. \end{array}$$ (28)

Recall in the *K* = 1 case each cascade is an independent event occurring with equal probability either side of 0. Hence, the cascade distribution, for fixed *q*, is simply the equally weighted mixture distribution of negative binomial components: a negative tail and a positive tail. The resulting distribution, *D*, is symmetric about 0 and therefore $\text{Var}\left(D\right)=E(D^{2})$. The moments of *D* are obtained using standard methods. In particular,

Var(D)=1220E2(Y1)+E2(Y2)+1222E(Y1-μ1)2+E(Y2-μ2)2=Var(X)+1+E(X)2. (29)

With *Y*_1_ = 1+*X*_1_ and *Y*_2_ = − 1 − *X*_2_, with *X*_1,2_(*n*, *p*) distributed negative binomial. For kurtosis we follow the same procedure as above.

ED4=12E4(Y1)+E4(Y2)+1242E2(Y1)E(Y1-μ1)2+E2(Y2)E(Y2-μ2)2+1243E(Y1)E(Y1-μ1)3+E(Y2)E(Y2-μ2)3+12E(Y1-μ1)4+E(Y2-μ1)4. (30)

The excess kurtosis expressed as a function of *q* is then,

$$\begin{array}{c} \text{Kurt}\left(D\right)=C\left(1-\frac{\left(q-2\right)q\left(a_{1}+a_{2}q\right)}{{\left(q-1\right)}^{2}}\right.\\ -\frac{4\left(q-2\right)q\left(q^{2}-2q-1\right)\left(a_{1}+a_{2}q\right)\left(q^{2}\left(a_{1}-2a_{2}-1\right)-2\left(a_{1}-1\right)q+a_{2}q^{3}-1\right)}{{\left(q-1\right)}^{8}}\\ -\frac{6\left(q-2\right)q\left(a_{1}+a_{2}q\right){\left(q^{2}\left(a_{1}-2a_{2}-1\right)-2\left(a_{1}-1\right)q+a_{2}q^{3}-1\right)}^{2}}{{\left(q-1\right)}^{8}}\\ \left.-\frac{\left(q-2\right)q\left(a_{1}+a_{2}q\right)\left(-3q^{2}\left(a_{1}-2a_{2}\right)+\left(6a_{1}+8\right)q-\left(3a_{2}+4\right)q^{3}+q^{4}+1\right)}{{\left(q-1\right)}^{8}}\right)-3 \end{array}$$ (31)

where

$$\begin{array}{c} C={\left({\left(\frac{\left(q-2\right)q\left(a_{1}+a_{2}q\right)}{{\left(q-1\right)}^{2}}-1\right)}^{2}-\frac{\left(q-2\right)q\left(a_{1}+a_{2}q\right)}{{\left(q-1\right)}^{4}}\right)}^{-2} \end{array}$$

### Power law distribution and Kolmogrov-Smirnov test

We use the discrete power law zeta distribution, which has density

$$\begin{array}{c} f\left(x\right)=x^{-\alpha }/\zeta \left(\alpha \right), \end{array}$$ (32)

where *ζ*(*α*) is the Riemann zeta function *ζ*(*α*) = ∑_*x*_ *x*^ − *α*^ with the sum over all integers *x*. The computation of the MLEs and Kolmogorov-Smirnov test statistics follow the procedures described in [62].

### Recovery of the implied volatility smile

We recover the implied volatility smile from quoted option prices using a numerical root search on the pricing formula for European call options [51]. Second, we use the simple empirical option pricing scheme outlined in [74] to compute the price an option via simulations of the probability distribution. From this we can again obtain the implied volatility from our model, and iterate the process until a reasonable fit is found to the market implied volatility.

## Discussion

We introduce two variants of a trader interaction model resulting in stochastic cascade processes and demonstrate a number of stylised facts of financial returns can be captured by incorporating the cascade processes into a simple financial market model. A novelty of the model is in its parametrisation by network coupling probability, which can be viewed as an order parameter for herd behaviour. Related research stresses the importance of heterogeneous trader time-scales in examining volatility clustering and long-memory patterns in asset price returns. While the analysis presented here is consistent with this view, our model allows for a study of such time scales as they arise from agents’ information accumulation process, and interaction with other agents. In the context of informational cascades, when agents are permitted, informationally, to be very far from instigating a trade (represented as accumulating information on an unbounded state-space), long-memory patters in asset return volatility are exhibited in situations where the distribution of firing thresholds amongst agents is left-skewed, resulting in a few relatively influential agents, and many more agents that have a higher propensity to herd.

## Supporting Information

S1 Dataset Market data price returns and implied volatility. A compressed file containing log-returns computed from empirical market data for General Electric at both daily, and intraday, frequencies. Dates or timestamps are included in the files where relevant, and only non-zero returns are used. A separate file containing the implied volatility market data for SPXpm European call options, as of 25 November 2014, is included.
(ZIP)

S1 Code Computer code used to simulate the cascade processes. A compressed file containing separate R and C++ files, together with a description file containing instructions for their use within the open source R programming environment.
(ZIP)
